# Associations of diverse diseases with work productivity among Japanese workers in the context of multimorbidity: additional analysis of a database study

**DOI:** 10.1093/joccuh/uiag033

**Published:** 2026-06-27

**Authors:** Takuya Maekawa, Kentaro Yamato, Norihiro Nakamichi, Masaya Takahashi, Ryotaro Ishii, Takeo Nakayama

**Affiliations:** Medical Affairs, Otsuka Pharmaceutical Co, Ltd, Shinagawa Grand Central Tower, 2-16-4 Konan, Minato-ku, Tokyo 108-8242, Japan; Medical Affairs, Otsuka Pharmaceutical Co, Ltd, Shinagawa Grand Central Tower, 2-16-4 Konan, Minato-ku, Tokyo 108-8242, Japan; Department of Public Health, Graduate School of Medicine, Juntendo University, Tokyo, Japan; Medical Affairs, Otsuka Pharmaceutical Co, Ltd, Shinagawa Grand Central Tower, 2-16-4 Konan, Minato-ku, Tokyo 108-8242, Japan; Research Center for Overwork-Related Disorders, National Institute of Occupational Safety and Health, Kawasaki, Japan; Department of Neurology, Kyoto Prefectural University of Medicine, Kyoto, Japan; Department of Health Informatics, Graduate School of Medicine and School of Public Health, Kyoto University, Kyoto, Japan

**Keywords:** burden of disease, productivity, real world data, Japan, comorbidities

## Abstract

**Objectives:**

Building on the previous descriptive study of work productivity across various diseases among Japanese workers, this additional analysis aimed to assess the associations between multimorbidity and work productivity, as well as independent associations between each disease and work productivity, accounting for coexisting diseases.

**Methods:**

From the original study population of the secondary dataset derived from health insurance claims and online surveys, including workers aged ≥19 years who completed the Work Productivity and Activity Impairment questionnaire–General Health (WPAI-GH) in an online survey in 2021, we analyzed participants aged <65 years (*n* = 30 953). The association of a WPAI score >0% with diseases count and each target disease adjusted for other diseases was explored using logistic regression, and the predicted probabilities of having a score >0% for each disease were derived.

**Results:**

The odds ratios (ORs) for having a WPAI score of >0% increased (OR > 1) per additional disease. Associated with higher odds of having a score >0% were, in males, migraine, depression, dysthymia, diarrheal disorders, and insomnia; and in females, migraine, insomnia, and atopic dermatitis. Femoral neck fracture, dysthymia, depression, migraine, and menopausal disorder in males, and migraine and ischemic heart disease in females were among the top diseases with high predicted probabilities.

**Conclusions:**

Each incremental disease was significantly associated with impaired work productivity. After adjusting for other comorbidities, work productivity impairment was associated with psychiatric disorders and migraines. These findings underscore the importance of optimal management of preexisting conditions and prevention of comorbidities.

## Teaser

**Key points**
 

**What is already known on this topic:** Having multiple coexisting diseases, that is, multimorbidity, is expected to have a cumulative adverse impact on work productivity; however, evidence among the working population remains limited. Our previous study provided an overview of work productivity and daily activity impairment across diverse diseases in a large working population in Japan; however, multimorbidity was not considered.
**What this study adds:** We demonstrated that both the number of diseases and specific conditions were associated with work productivity and daily activities among workers in Japan, with sex- and age-specific variations in the patterns of associated diseases, after adjusting for comorbidities.
**How this study might affect research, practice, or policy:** These findings highlight the importance of optimal, early, and individually tailored management of preexisting conditions and prevention of the development of additional diseases. From an occupational health perspective, attention should also be paid to common diseases that individually have a modest impact, but may collectively affect workplace productivity and sustainability.

## 1. Introduction

Multimorbidity, the coexistence of multiple diseases within a person, poses a critical challenge to the global health care system, precipitating a rise in mortality,^1^ demands on health care resources,^2^ and economic burden.^3^ Although often regarded as an issue confined to older adults, multimorbidity also extends to younger generations.^4^^,^^5^ The prevalence of multimorbidity in individuals younger than 65 years is reported to be comparable to that in older populations, with frequent coexistence of physical and mental diseases.^4^ This suggests that multimorbidity may be highly relevant to the working-age population. However, evidence of its specific impact in this population remains limited.

A review on multimorbidity and work productivity has summarized the available studies,^6^ albeit few, including those conducted in Finnish workers.^7^^,^^8^ These studies indicated that psychiatric disorders, particularly depression, are among the major contributors to work disability, and that their coexistence with other chronic conditions further increases the risk of work disability and hinders return to work.^7^^,^^8^ One of the few studies that evaluated the influence of multimorbidity on work, in terms of not only absenteeism (absence from work) but also presenteeism (reduced performance at work), reported that concurrent chronic conditions were associated with a marked increase in both absenteeism and presenteeism rates, indicating poorer work productivity, as well as an elevated risk of work-related critical incidents.^9^ The study also demonstrated that the impact of multimorbidity far exceeded that of a single condition.^9^

However, previous research has been limited by the fact that it focused on single diseases without adjusting for comorbidities. Given that some diseases, such as depression and insomnia,^10^ frequently co-occur, the previous findings could not establish whether the observed disease burden reflected a specific primary disease or a combination of diseases. Our previous study using a large-scale database to describe the proportion of workers experiencing work productivity and activity impairment (WPAI) across various diseases had the same limitation.^11^ Thus, there remains limited evidence regarding independent associations between individual diseases and work productivity impairment in the context of multimorbidity.

Therefore, we conducted an additional analysis to assess the associations between multimorbidity and WPAI, in terms of the number of diseases and conditions, and to assess the independent association of each disease with the WPAI, accounting for coexisting diseases and conditions.

## 2. Methods

### 2.1 Study overview and data source

This was an additional analysis of a retrospective descriptive study that used secondary data from health insurance claims and survey responses. The details of these methods have been reported previously.^11^ For this additional analysis, we used the same dataset as in the original study but focused on a subset of participants aged <65 years, as described below.

The database used for the original and this additional analysis is maintained by DeSC Healthcare, Inc, Tokyo, Japan (DeSC), which obtains data on health insurance claims and survey responses from multiple insurers and compiles them into a large-scale database. The surveys are conducted regularly by DeSC via their original health promotion application, Kencom®, and are not administered by the investigators. We utilized data from the enrollees of society-managed employment-based health insurance associations, one of the national health insurance schemes in Japan, covering employees of large enterprises and their family members. We used health insurance claims data generated between January and December 2021, and data from the survey conducted in June and December of the same year. In the case of both survey data being available for a participant, only the earliest response data (June) were used. All data were linked and anonymized by DeSC before being provided to the study investigators.

### 2.2 Study population

The original study included the participants who were insured by the insurance associations consenting to the data usage (who were Kencom® registrants), who responded to either round of the survey, who were ≥19 years old at the time of the survey, who were self-reported current workers, and who completed the WPAI questionnaire–General Health (WPAI-GH). Those who self-reported as students, were unemployed, or had multiple sets of response data within the same survey round were excluded. Of the original analysis set of 31 540 participants, this additional analysis included participants aged <65 years (*n* = 30 953).

### 2.3 Ethics statement

This additional analysis used the same anonymized secondary dataset as in the original study. The original study was approved by the independent Ethics Committee of Otsuka Pharmaceutical Co, Ltd (approval no. 231004). As the data were anonymized, no additional informed consent was required for the original study.

### 2.4 Definitions

#### 2.4.1 Background characteristics

From the claims data (including insurance enrollment records), we assessed the sex, age, and disease, which are defined in the following section. From the survey data, we assessed job categories and residential areas.

#### 2.4.2 Target disease

We identified and analyzed the same set of 44 diseases in the claims data as in the original study. These diseases were defined using the corresponding International Classification of Diseases, 10th Revision (ICD-10) codes, which are detailed in our previous paper.^11^ Principally, these diseases were selected to reflect the leading causes of years lived with disability globally.^12^ The diseases included were key health problems related to occupational health,^13^ those related to working women,^14^ and the top 5 common cancers,^15^ according to official Japanese governmental publications.

The number of diseases was defined as the number of target diseases identified in each participant between January and December, 2021.

#### 2.4.3 Work productivity

Impairment in work productivity and daily life during the last 7 days was assessed based on survey data from the WPAI-GH.^16^ The WPAI-GH yields 4 domain scores of work productivity: absenteeism (work hours missed), presenteeism (impairment while working), total work impairment (overall work productivity impairment due to absenteeism and presenteeism), and total activity impairment (impairment in daily life). The score is expressed as a percentage, with higher values indicating greater impairment.

### 2.5 Statistical analysis

Analyses were primarily conducted for subgroups defined by the combination of sex (male and female) and age categories (≤29, 30-39, 40-49, 50-64 years, and all ages). Background characteristics were summarized, with categorical variables presented as numbers (percentages) and continuous variables presented as mean (SD) and median (first to third quartile).

The association between the number of target diseases and the WPAI was examined using logistic regression analysis with the number of diseases as the independent variable. As a dependent variable, we used the dichotomized WPAI-GH domain score (0% or >0%) given that many participants scored 0% in the original analysis. The frequencies of comorbidity combination patterns for men and women were summarized and visualized using UpSet plots.

We also explored the association between each target disease and the WPAI using a multivariate logistic regression model. The presence or absence of each of the 44 diseases was treated as the independent variable, with the absence of the corresponding disease as a reference and the dichotomized WPAI-GH domain score (0% or >0%) as the dependent variable. To ensure stable estimates, diseases were excluded from the model if contingency tables with dichotomized WPAI-GH scores contained zero cells. Odds ratios (ORs) with 95% CIs and *P* values were estimated. This additional analysis was exploratory in nature; *P* values were provided for reference only and not for confirmatory interpretation. Moreover, to assess the robustness of the binary logistic regression analysis for the association between each disease and WPAI, sensitivity analyses using ordinal logistic regression models were conducted.

The adjusted predicted probabilities of having a WPAI-GH score >0% were also calculated using the logistic regression model. The predicted probability of each target disease was plotted against the period prevalence of each disease. The period prevalence was calculated in the same manner as previously described, that is, the number of participants with a corresponding ICD-10 code recorded between January and December 2021 divided by the number of participants in each subgroup.

As inclusion in the analysis required completion of the WPAI-GH, no missing data were imputed. SAS software (version 9.4; SAS Institute, Cary, NC, USA) was used for all analyses.

## 3. Results

### 3.1 Participant characteristics

Table 1 summarizes the demographic characteristics of the participants included in this additional analysis (*n* = 30 953), consisting of 72.0% males, with a mean (SD) age of 47.8 (9.9) years (median [first, third quartile]: 49 [41, 55]). For both males and females, the proportion of professional/technical and marketing workers tended to be higher among those aged <40 years relative to those aged ≥40 years. Clerical workers were also common, accounting for the largest proportion of females. Meanwhile, the proportion of workers at managerial positions was higher in those aged ≥40 years, relative to younger subgroups. The largest proportions of participants resided in Kanagawa, Tokyo, Hyogo, Osaka, and Saitama. The mean number of diseases increased slightly with age. Women had more diseases than men, with higher mean numbers across age subgroups (range in women: 2.9-3.8; range in men: 1.8-3.2).

**Table 1:** Background characteristics of analysis population by sex and age subgroups.

	Total	Male					Female				
		All ages	≤29 y	30-39 y	40-49 y	50-64 y	All ages	≤29 y	30-39 y	40-49 y	50-64 y
	*n* (%)	*n* (%)	*n* (%)	*n* (%)	*n* (%)	*n* (%)	*n* (%)	*n* (%)	*n* (%)	*n* (%)	*n* (%)
**Overall, *n***	30 953	22 292	1083	3083	6055	12 071	8661	768	1501	3042	3350
**Job category** ^**a**^											
**Professional/technical workers**	7681 (24.8)	6632 (29.8)	465 (42.9)	1431 (46.4)	1799 (29.7)	2937 (24.3)	1049 (12.1)	179 (23.3)	259 (17.3)	297 (9.8)	314 (9.4)
**Managerial positions**	5609 (18.1)	5349 (24.0)	6 (0.6)	52 (1.7)	1264 (20.9)	4027 (33.4)	260 (3.0)	2 (0.3)	1 (0.1)	95 (3.1)	162 (4.8)
**Clerical workers**	7883 (25.5)	3245 (14.6)	129 (11.9)	467 (15.1)	771 (12.7)	1878 (15.6)	4638 (53.6)	354 (46.1)	803 (53.5)	1775 (58.3)	1706 (50.9)
**Marketing workers**	2812 (9.1)	2255 (10.1)	201 (18.6)	419 (13.6)	549 (9.1)	1086 (9.0)	557 (6.4)	111 (14.5)	158 (10.5)	150 (4.9)	138 (4.1)
**Sales workers**	233 (0.8)	102 (0.5)	6 (0.6)	11 (0.4)	28 (0.5)	57 (0.5)	131 (1.5)	3 (0.4)	14 (0.9)	39 (1.3)	75 (2.2)
**Transportation and communication workers**	57 (0.2)	41 (0.2)	5 (0.5)	5 (0.2)	10 (0.2)	21 (0.2)	16 (0.2)	0 (0.0)	2 (0.1)	9 (0.3)	5 (0.1)
**Security workers**	90 (0.3)	89 (0.4)	6 (0.6)	13 (0.4)	29 (0.5)	41 (0.3)	1 (0.0)	0 (0.0)	0 (0.0)	0 (0.0)	1 (0.0)
**Skilled trades**	1944 (6.3)	1795 (8.1)	159 (14.7)	320 (10.4)	691 (11.4)	625 (5.2)	149 (1.7)	29 (3.8)	25 (1.7)	50 (1.6)	45 (1.3)
**Agriculture, forestry, and fisheries**	36 (0.1)	19 (0.1)	1 (0.1)	3 (0.1)	6 (0.1)	9 (0.1)	17 (0.2)	0 (0.0)	0 (0.0)	8 (0.3)	9 (0.3)
**Service (qualification required)**	209 (0.7)	79 (0.4)	3 (0.3)	11 (0.4)	25 (0.4)	40 (0.3)	130 (1.5)	1 (0.1)	14 (0.9)	46 (1.5)	69 (2.1)
**Service (qualification not required)**	963 (3.1)	543 (2.4)	27 (2.5)	62 (2.0)	173 (2.9)	281 (2.3)	420 (4.8)	22 (2.9)	62 (4.1)	152 (5.0)	184 (5.5)
**Others**	3436 (11.1)	2143 (9.6)	75 (6.9)	289 (9.4)	710 (11.7)	1069 (8.9)	1293 (14.9)	67 (8.7)	163 (10.9)	421 (13.8)	642 (19.2)
**Prefecture of residence** ^**a**^ ^**,**^ ^**b**^											
**Kanagawa**	5544 (17.9)	4104 (18.4)	156 (14.4)	500 (16.2)	960 (15.9)	2488 (20.6)	1440 (16.6)	139 (18.1)	225 (15.0)	485 (15.9)	591 (17.6)
**Tokyo**	3995 (12.9)	2723 (12.2)	152 (14.0)	349 (11.3)	675 (11.1)	1547 (12.8)	1272 (14.7)	144 (18.8)	263 (17.5)	416 (13.7)	449 (13.4)
**Hyogo**	3197 (10.3)	2430 (10.9)	128 (11.8)	358 (11.6)	625 (10.3)	1319 (10.9)	767 (8.9)	59 (7.7)	132 (8.8)	256 (8.4)	320 (9.6)
**Osaka**	1894 (6.1)	1292 (5.8)	82 (7.6)	209 (6.8)	335 (5.5)	666 (5.5)	602 (7.0)	56 (7.3)	124 (8.3)	199 (6.5)	223 (6.7)
**Saitama**	1861 (6.0)	1404 (6.3)	49 (4.5)	171 (5.5)	384 (6.3)	800 (6.6)	457 (5.3)	37 (4.8)	75 (5.0)	163 (5.4)	182 (5.4)
**Number of diseases, continuous** ^**c**^											
**Mean**	3.0	2.8	1.8	2.2	2.4	3.2	3.4	2.9	3.1	3.4	3.8
**SD**	2.5	2.4	1.8	2.0	2.1	2.5	2.7	2.1	2.4	2.7	2.8
**Median**	3.0	2.0	1.0	2.0	2.0	3.0	3.0	3.0	3.0	3.0	3.0
**Q1-Q3**	1.0-4.0	1.0-4.0	0.0-3.0	1.0-3.0	1.0-4.0	1.0-5.0	1.0-5.0	1.0-4.0	1.0-4.0	1.0-5.0	2.0-5.0
**Number of diseases, category** ^**c**^											
**0**	4537 (14.7)	3636 (16.3)	299 (27.6)	609 (19.8)	1156 (19.1)	1572 (13.0)	901 (10.4)	89 (11.6)	157 (10.5)	315 (10.4)	340 (10.1)
**1**	6004 (19.4)	4548 (20.4)	252 (23.3)	765 (24.8)	1428 (23.6)	2103 (17.4)	1456 (16.8)	142 (18.5)	281 (18.7)	551 (18.1)	482 (14.4)
**2**	4760 (15.4)	3484 (15.6)	200 (18.5)	557 (18.1)	1002 (16.5)	1725 (14.3)	1276 (14.7)	136 (17.7)	250 (16.7)	465 (15.3)	425 (12.7)
**3**	4914 (15.9)	3494 (15.7)	167 (15.4)	478 (15.5)	915 (15.1)	1934 (16.0)	1420 (16.4)	140 (18.2)	251 (16.7)	493 (16.2)	536 (16.0)
**4**	3640 (11.8)	2511 (11.3)	77 (7.1)	287 (9.3)	642 (10.6)	1505 (12.5)	1129 (13.0)	116 (15.1)	207 (13.8)	382 (12.6)	424 (12.7)
**5**	2525 (8.2)	1704 (7.6)	44 (4.1)	187 (6.1)	354 (5.8)	1119 (9.3)	821 (9.5)	64 (8.3)	133 (8.9)	275 (9.0)	349 (10.4)
**6**	1699 (5.5)	1140 (5.1)	23 (2.1)	85 (2.8)	244 (4.0)	788 (6.5)	559 (6.5)	38 (4.9)	85 (5.7)	190 (6.2)	246 (7.3)
**7**	1193 (3.9)	776 (3.5)	10 (0.9)	53 (1.7)	155 (2.6)	558 (4.6)	417 (4.8)	21 (2.7)	61 (4.1)	129 (4.2)	206 (6.1)
**8**	695 (2.2)	432 (1.9)	3 (0.3)	33 (1.1)	69 (1.1)	327 (2.7)	263 (3.0)	9 (1.2)	30 (2.0)	103 (3.4)	121 (3.6)
**9**	427 (1.4)	256 (1.1)	5 (0.5)	11 (0.4)	56 (0.9)	184 (1.5)	171 (2.0)	6 (0.8)	21 (1.4)	57 (1.9)	87 (2.6)
**10**	244 (0.8)	155 (0.7)	1 (0.1)	8 (0.3)	15 (0.2)	131 (1.1)	89 (1.0)	4 (0.5)	13 (0.9)	24 (0.8)	48 (1.4)
**11**	134 (0.4)	68 (0.3)	0 (0.0)	5 (0.2)	7 (0.1)	56 (0.5)	66 (0.8)	1 (0.1)	3 (0.2)	18 (0.6)	44 (1.3)
**12**	93 (0.3)	53 (0.2)	1 (0.1)	3 (0.1)	9 (0.1)	40 (0.3)	40 (0.5)	1 (0.1)	3 (0.2)	18 (0.6)	18 (0.5)
**13**	41 (0.1)	16 (0.1)	0 (0.0)	2 (0.1)	2 (0.0)	12 (0.1)	25 (0.3)	1 (0.1)	2 (0.1)	10 (0.3)	12 (0.4)
**14**	21 (0.1)	10 (0.0)	1 (0.1)	0 (0.0)	1 (0.0)	8 (0.1)	11 (0.1)	0 (0.0)	2 (0.1)	5 (0.2)	4 (0.1)
**15**	16 (0.1)	7 (0.0)	0 (0.0)	0 (0.0)	0 (0.0)	7 (0.1)	9 (0.1)	0 (0.0)	1 (0.1)	4 (0.1)	4 (0.1)
**16**	6 (0.0)	1 (0.0)	0 (0.0)	0 (0.0)	0 (0.0)	1 (0.0)	5 (0.1)	0 (0.0)	1 (0.1)	1 (0.0)	3 (0.1)
**17**	3 (0.0)	1 (0.0)	0 (0.0)	0 (0.0)	0 (0.0)	1 (0.0)	2 (0.0)	0 (0.0)	0 (0.0)	1 (0.0)	1 (0.0)
**18**	0 (0.0)	0 (0.0)	0 (0.0)	0 (0.0)	0 (0.0)	0 (0.0)	0 (0.0)	0 (0.0)	0 (0.0)	0 (0.0)	0 (0.0)
**19**	1 (0.0)	0 (0.0)	0 (0.0)	0 (0.0)	0 (0.0)	0 (0.0)	1 (0.0)	0 (0.0)	0 (0.0)	1 (0.0)	0 (0.0)
**20**	0 (0.0)	0 (0.0)	0 (0.0)	0 (0.0)	0 (0.0)	0 (0.0)	0 (0.0)	0 (0.0)	0 (0.0)	0 (0.0)	0 (0.0)

Abbreviation: Q, quartile.

Percentages may not sum to 100% because of rounding.

^a^Defined based on the survey response data.

^b^Top 5 areas in the total analysis population are listed.

^c^Defined based on the claims data.

### 3.2 Association of disease number with WPAI

**Figure 1:**
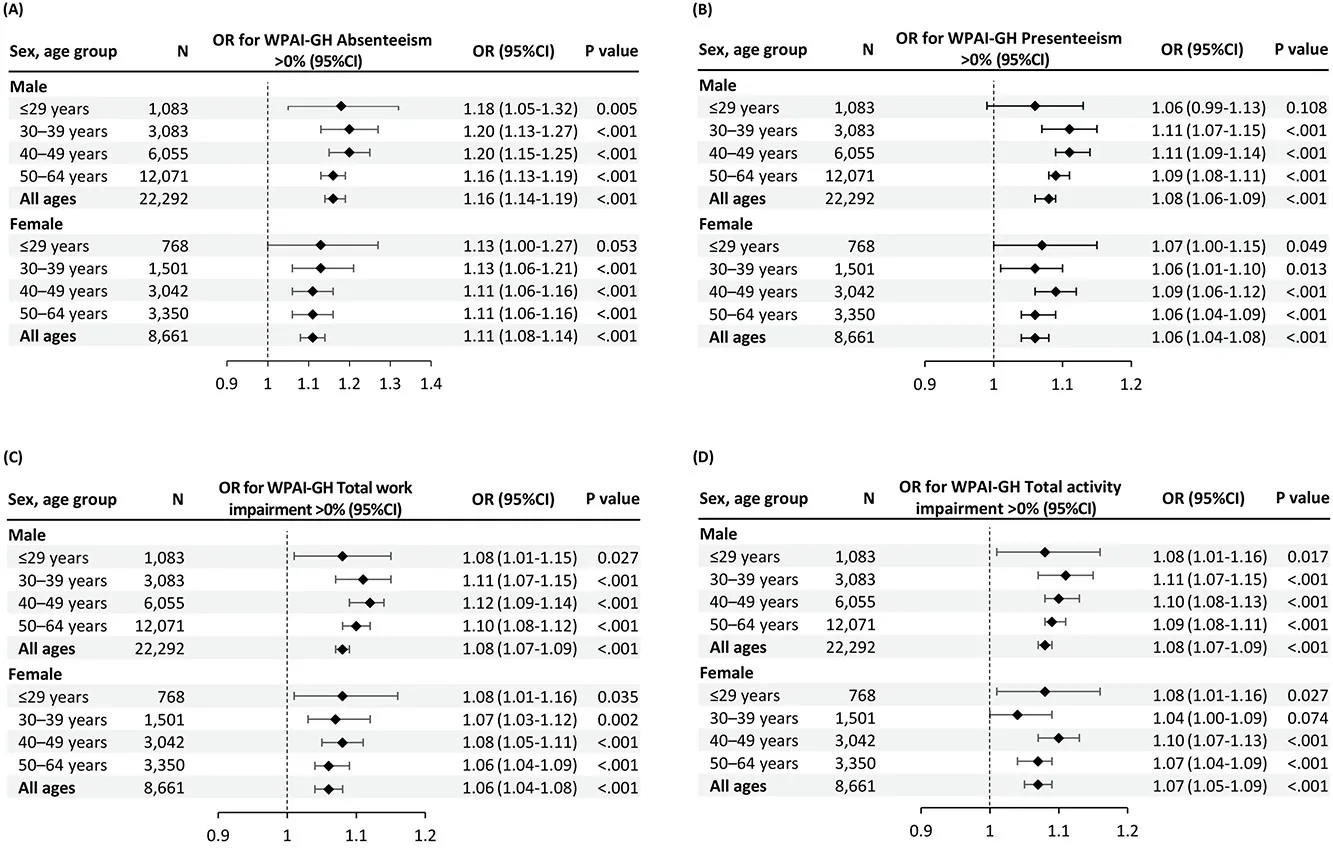
Odds ratio for a WPAI-GH score of >0% per additional disease, by domain: (A) absenteeism, (B) presenteeism, (C) total work impairment, and (D) total activity impairment. OR, odds ratio; WPAI-GH, Work Productivity and Activity Impairment questionnaire–General Health.

Figure 1 shows the ORs for having a WPAI-GH score >0% per additional disease. ORs for having a WPAI-GH score >0% consistently exceeded 1, ranging from 1.04 to 1.20, across all domains and sex and age subgroups. Statistical significance was observed in the all-age combined groups of males and females, and in the majority of age-specific subgroups.

The frequencies of comorbidity combination patterns, including patterns of a single disease, in males and females are shown in Figures S1 and S2, respectively. Allergic rhinitis alone was the most frequent pattern observed in both males (*n* = 1110) and females (*n* = 407). The most common pattern of coexisting diseases was hypertension and hyperlipidemia (*n* = 344) in males, and allergic rhinitis and diseases of the esophagus, stomach, and duodenum (*n* = 67) in females. Oral-related diseases, sensory organ diseases, and disorders of refraction and accommodation were excluded because preventive oral care and contact lens prescriptions, which are recorded for these diseases in the insurance claims data, were considered extremely common and may obscure the pattern of comorbidity combinations.

### 3.3 Association of each disease with WPAI

**Figure 2:**
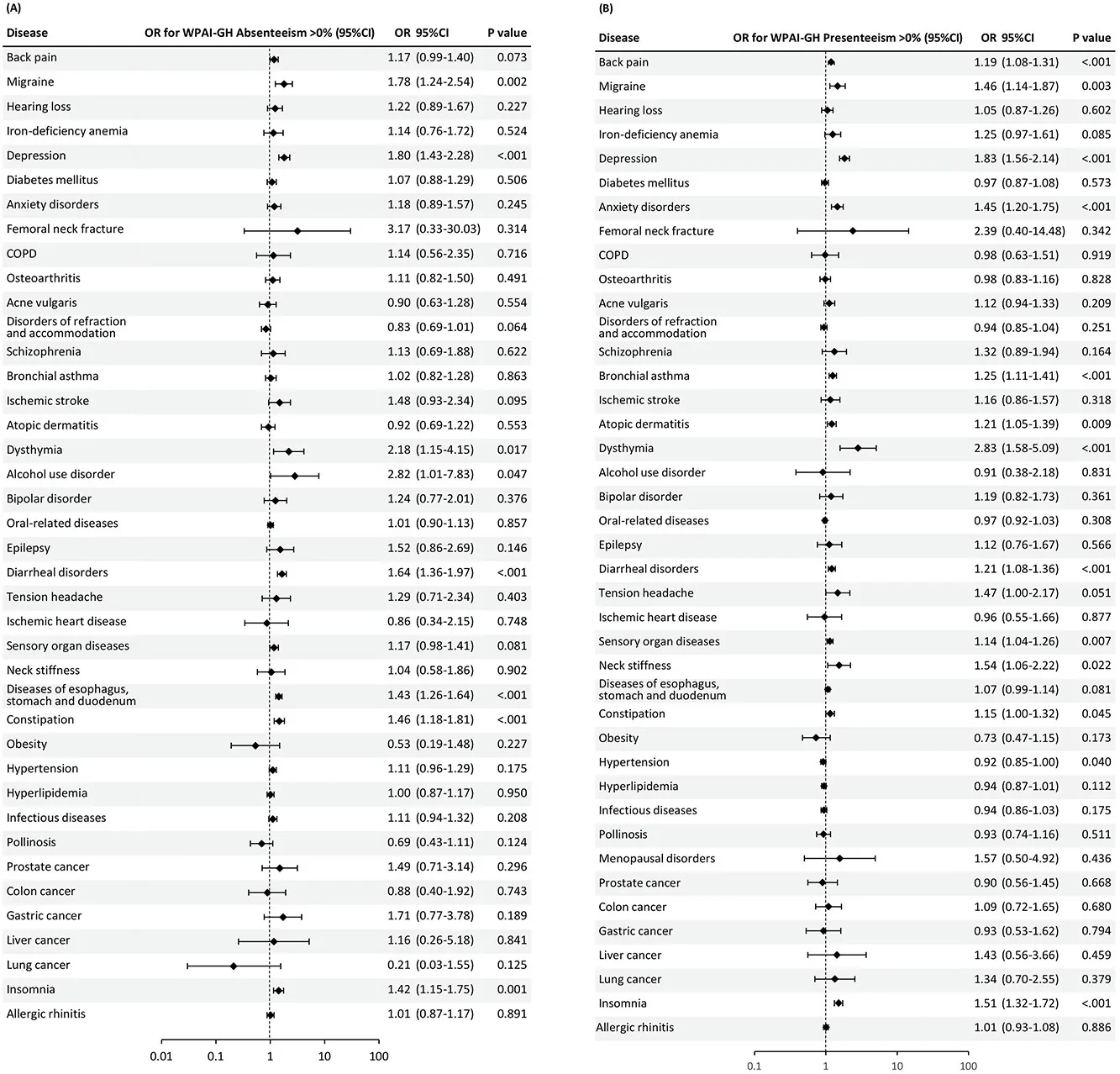

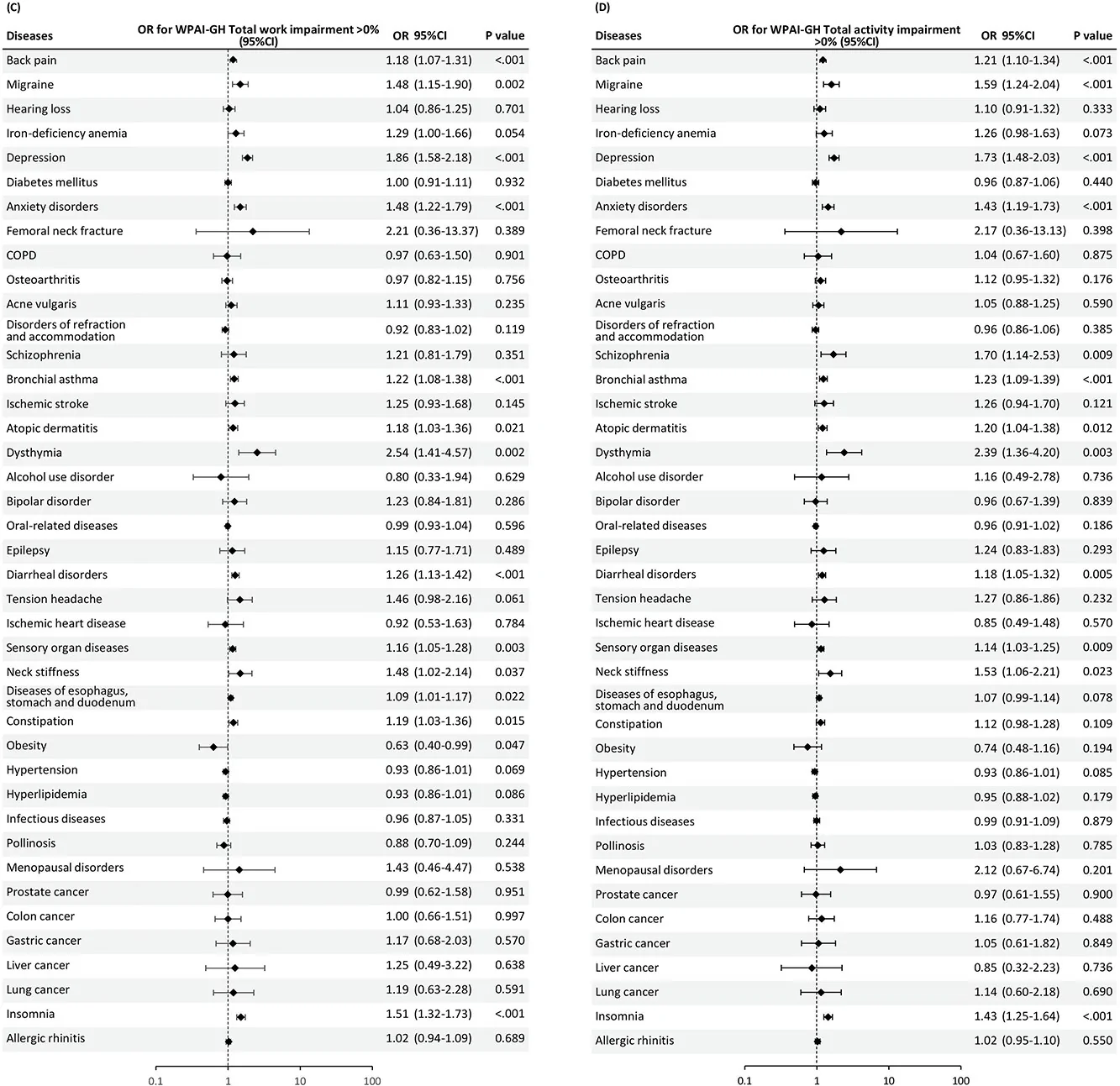
Odds ratio for a WPAI-GH score of >0% of each target disease in males by domain: (A) absenteeism, (B) presenteeism, (C) total work impairment, and (D) total activity impairment. Of the 44 target diseases, the following were excluded from each model because contingency tables with the dichotomized WPAI-GH score contained zero cells: menstrual disorders, menopausal disorders, uterine cancer, and breast cancer in absenteeism (A); menstrual disorders, uterine cancer, and breast cancer in presenteeism (B), total work impairment (C), and total activity impairment (D). COPD, chronic obstructive pulmonary disease; OR, odds ratio; WPAI-GH, Work Productivity and Activity Impairment questionnaire–General Health.

Figure 2 shows the ORs for WPAI-GH scores >0% for the target diseases in males. Among the diseases included in the model, migraine, depression, dysthymia, diarrheal disorders, and insomnia were significantly associated with increased odds (OR > 1) of having a score >0% across all domains. Several other diseases were significantly associated with higher odds across multiple domains. Back pain, anxiety disorders, bronchial asthma, atopic dermatitis, sensory organ diseases, and neck stiffness were significantly associated with presenteeism, total work impairment, and total activity impairment but not with absenteeism. Constipation and diseases of the esophagus, stomach, and duodenum also showed significantly greater odds in multiple, but not all, domains: constipation in absenteeism, presenteeism, and total work impairment, and diseases of the esophagus, stomach, and duodenum in absenteeism and total work impairment. In contrast, alcohol use disorder and schizophrenia were associated with significantly greater odds in only a single domain, absenteeism and total activity impairment, respectively.

**Figure 3:**
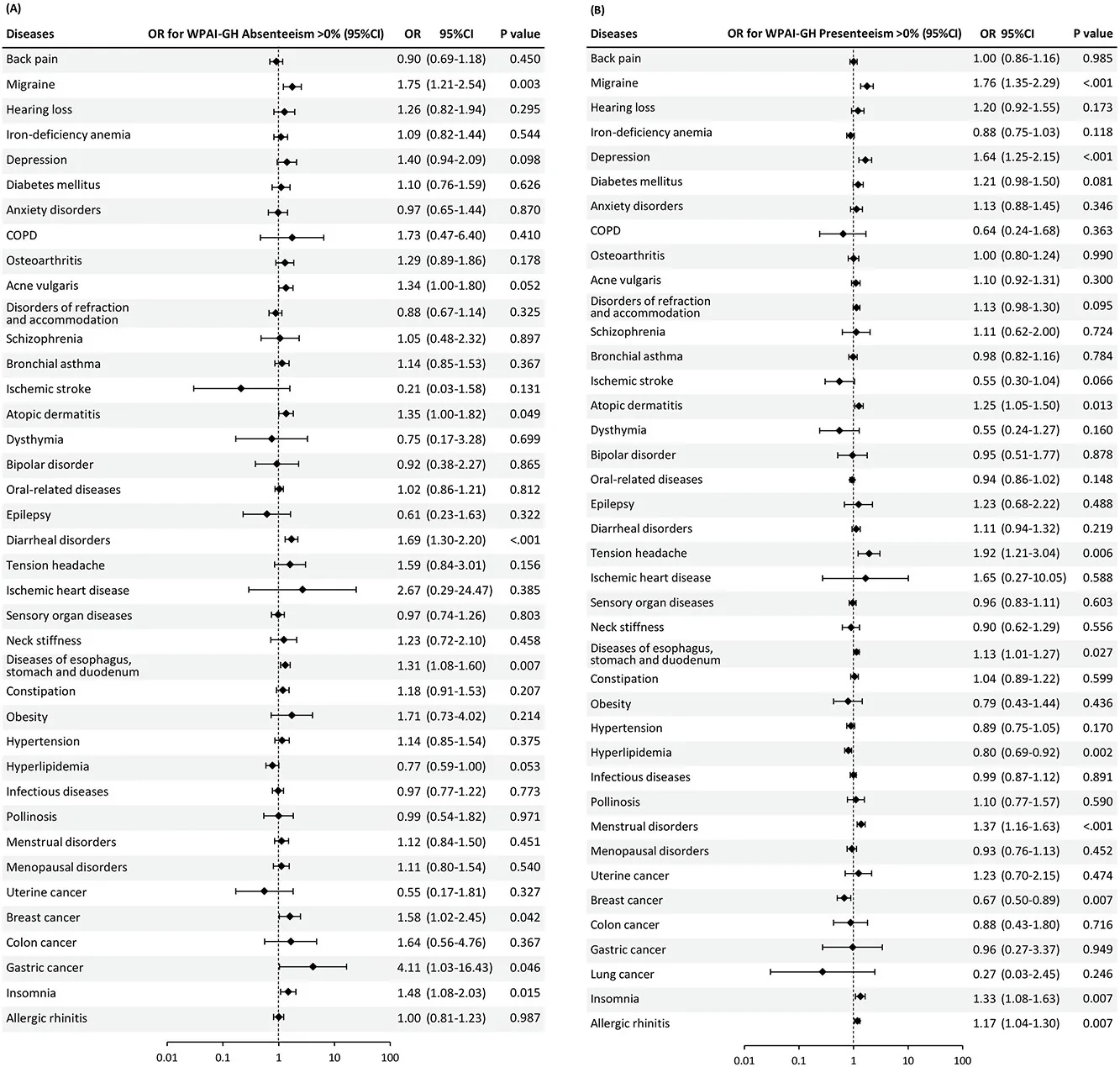

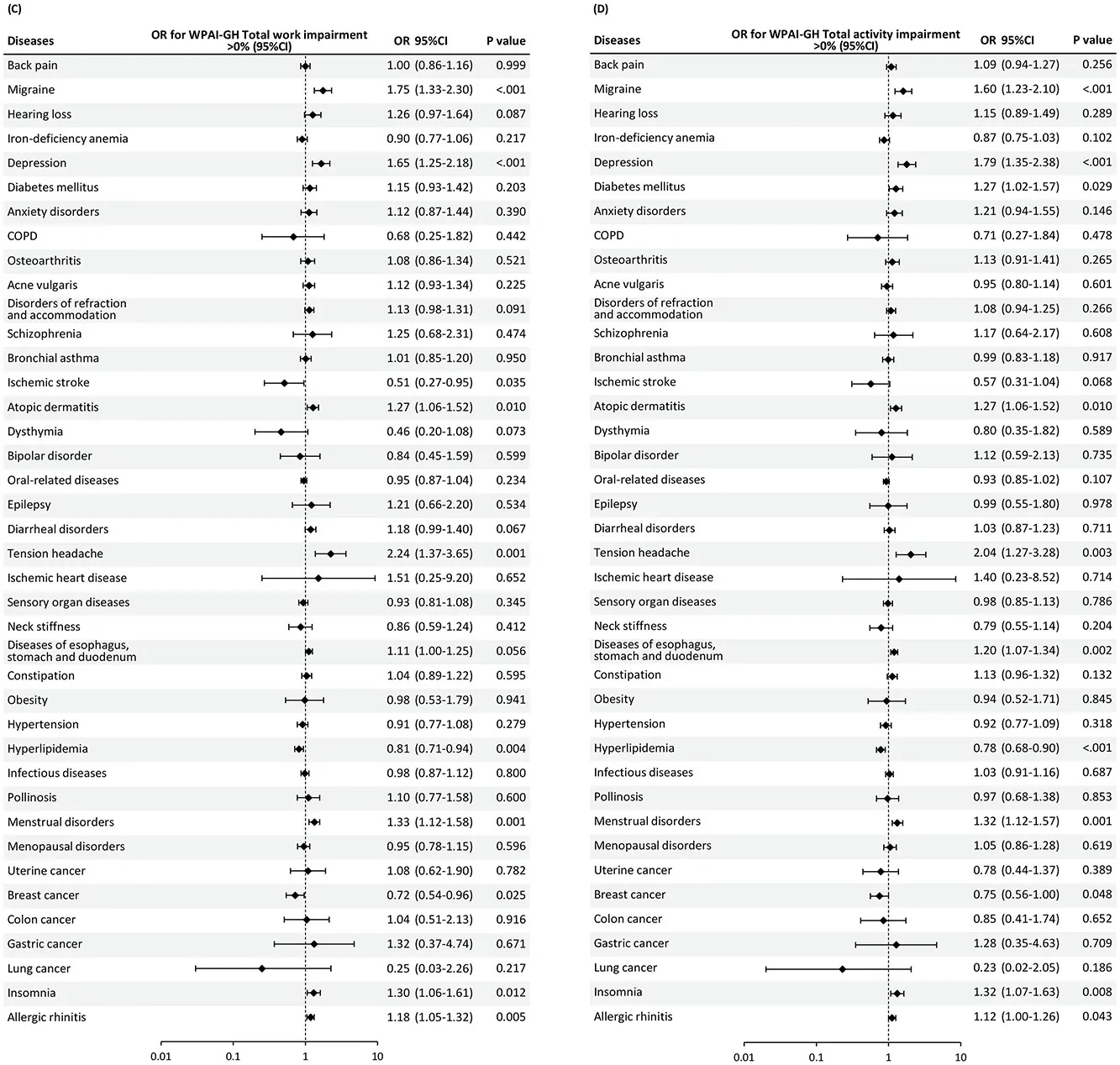
Odds ratio for a WPAI-GH score of >0% of each target disease in females by domain: (A) absenteeism, (B) presenteeism, (C) total work impairment, and (D) total activity impairment. Of the 44 target diseases, the following were excluded from each model because contingency tables with the dichotomized WPAI-GH score contained zero cells: femoral neck fracture, alcohol use disorder, prostate cancer, liver cancer, and lung cancer in absenteeism (A); femoral neck fracture, alcohol use disorder, prostate cancer, and liver cancer in presenteeism (B), total work impairment (C), and total activity impairment (D). COPD, chronic obstructive pulmonary disease; OR, odds ratio; WPAI-GH, Work Productivity and Activity Impairment questionnaire–General Health.

Among females, as among males, migraine and insomnia were significantly associated with increased odds (OR > 1) of having a WPAI-GH score >0% across all domains (Figure 3) as well as atopic dermatitis, which was not found for absenteeism among males. Depression, tension headaches, menstrual disorders, and allergic rhinitis showed significantly increased odds in multiple domains: presenteeism, total work impairment, and total activity impairment, but not in absenteeism. By contrast, patients with diarrheal disorders, breast cancer, and gastric cancer showed significantly increased odds only in absenteeism, and diabetes mellitus only in total activity impairment. In absenteeism, the OR was highest for gastric cancer (OR 4.11; 95% CI, 1.03-16.43); while in other domains, tension headaches showed the highest odds, approximately doubled compared with the reference.

The sensitivity analyses using ordinal logistic regression models yielded results generally consistent with those from the corresponding binary logistic regression models (Tables S1 and S2).

### 3.4 Predicted probability of WPAI score >0%

**Figure 4:**
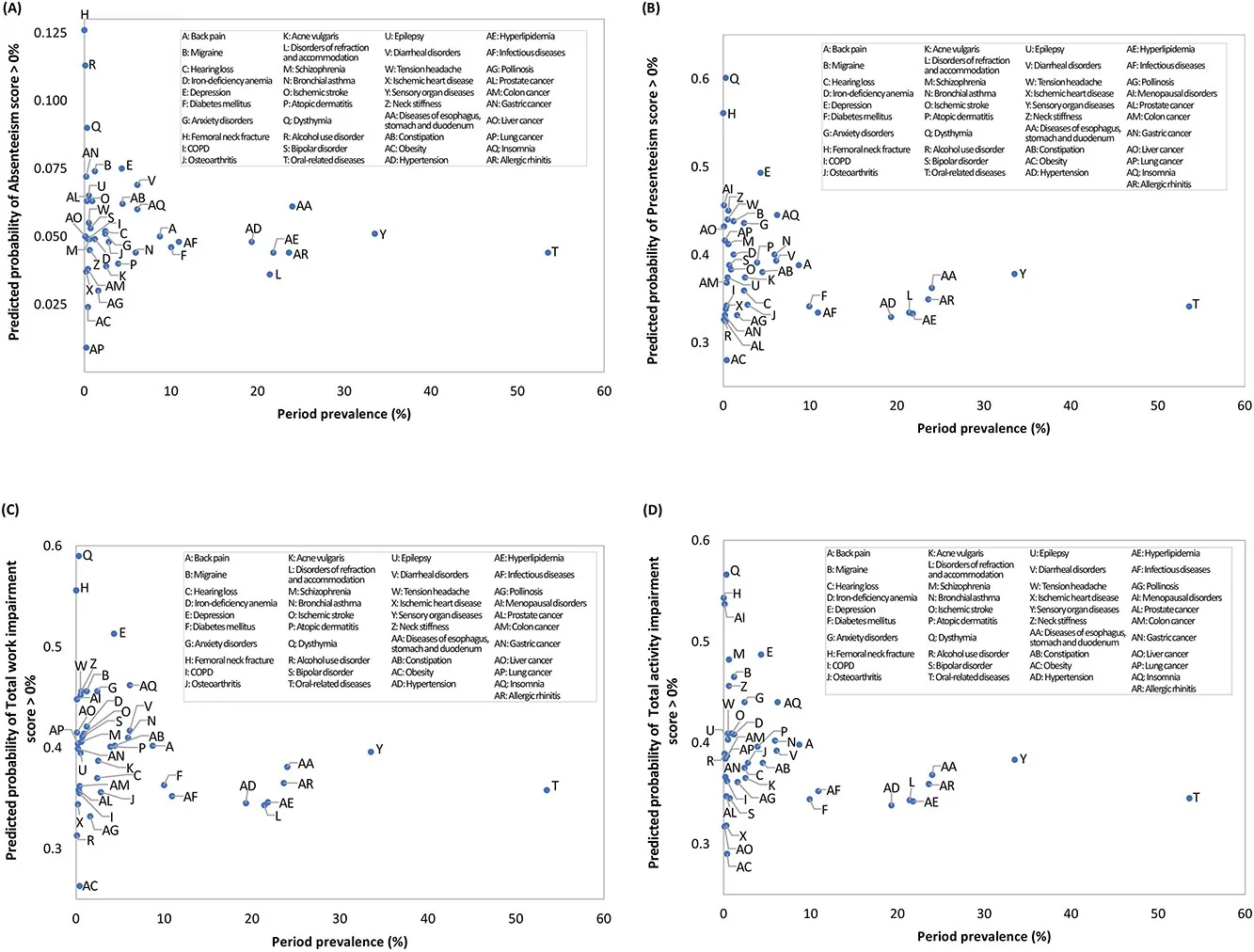
Scatter plot of predicted probability of a WPAI-GH score of >0% of each disease by period prevalence in males: (A) absenteeism, (B) presenteeism, (C) total work impairment, and (D) total activity impairment. Of the 44 target diseases, the following were excluded from each model because contingency tables with the dichotomized WPAI-GH score contained zero cells: menstrual disorders, menopausal disorders, uterine cancer, and breast cancer in absenteeism (A); menstrual disorders, uterine cancer, and breast cancer in presenteeism (B), total work impairment (C), and total activity impairment (D). COPD, chronic obstructive pulmonary disease; WPAI-GH, Work Productivity and Activity Impairment questionnaire–General Health.

**Figure 5:**
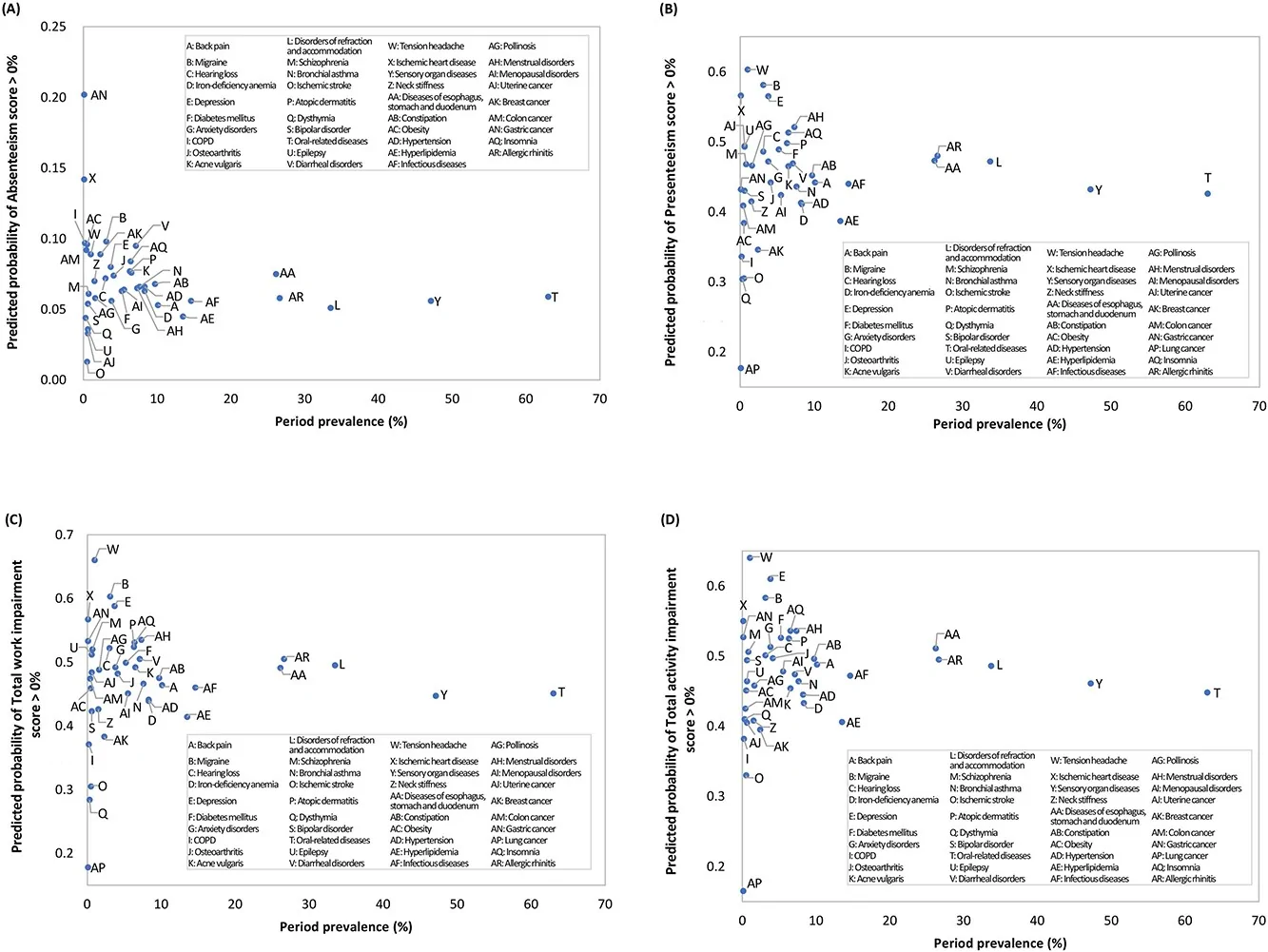
Scatter plot of predicted probability of a WPAI-GH score of >0% of each disease by period prevalence in females: (A) absenteeism, (B) presenteeism, (C) total work impairment, and (D) total activity impairment. Of the 44 target diseases, the following were excluded from each model because contingency tables with the dichotomized WPAI-GH score contained zero cells: femoral neck fracture, alcohol use disorder, prostate cancer, liver cancer, and lung cancer in absenteeism (A); femoral neck fracture, alcohol use disorder, prostate cancer, and liver cancer in presenteeism (B), total work impairment (C), and total activity impairment (D). COPD, chronic obstructive pulmonary disease; WPAI-GH, Work Productivity and Activity Impairment questionnaire–General Health.

Figures 4 and 5 plot the predicted probabilities of having a WPAI-GH score >0% for each target disease against the period prevalence among males and females, respectively. Among males, femoral neck fracture, dysthymia, depression, migraine, and menopausal disorder were among the top 5 diseases with the highest predicted probabilities across multiple domains. Particularly, dysthymia and femoral neck fracture had the highest and second-highest predicted probabilities (0.5-0.6), respectively, in 3 domains (presenteeism, total work impairment, and total activity impairment). The remaining top 5 positions were filled by different diseases by domain; for example, alcohol use disorder in absenteeism, neck stiffness in presenteeism, insomnia in total work impairment, and schizophrenia in total activity impairment.

Among females, migraine and ischemic heart disease consistently ranked among the top 5 diseases with the highest predicted probabilities across all domains. The remaining 3 positions in the top 5 varied: in the absenteeism domain they were occupied by gastric cancer, chronic obstructive pulmonary disease, and obesity, while in the other domains they were filled by tension headache, depression, and menstrual disorders. Notably, tension headache ranked first in the latter domains (presenteeism, total work impairment, and total activity impairment) with a predicted probability exceeding 0.6.

Focusing on diseases that were significantly associated with increased odds of having WPAI-GH scores >0% in the model for each sex group, Figures S3 and S4 show the predicted probabilities of having the WPAI-GH score >0% for these diseases by age subgroups (≤29, 30-39, 40-49, and 50-64 years old) in males and females, respectively. Age-related variations were noted in dysthymia, peaking in men 40-49 years old. A temporary increase in tension headaches was observed in women of the same age group. In females, the predicted probabilities of having the WPAI-GH score >0% appeared higher in the youngest subgroup of ≤29 years old than the older subgroups for depression, diabetes, and migraine.

## 4. Discussion

Building upon a previous descriptive study, we further evaluated the association between the number of diseases and each of the 44 target diseases using the WPAI in the context of multimorbidity. The results demonstrated that the odds of having a WPAI-GH score of >0% increased for each additional disease. Logistic regression analysis accounting for other diseases showed elevated odds of having all domain scores of >0% for migraine, depression, dysthymia, diarrheal disorders, and insomnia in males, and for migraine, insomnia, and atopic dermatitis in females. When converted to predicted probabilities, some diseases showed consistently high predicted probabilities across the domains, that is, femoral neck fracture, dysthymia, depression, migraine, and menopausal disorder in males, and migraine and ischemic heart disease in females.

Using a large-scale dataset derived from employment-based health insurance associations, the present analysis may reflect certain characteristics of the current Japanese labor market. For example, in the present results, clerical workers were relatively prevalent across the age groups, while professional/technical workers accounted for a larger proportion in younger generations than other age groups, in line with the Japanese national labor force survey.^17^ A higher percentage of managerial positions among males ≥40 years old may also reflect the general trend of age-related changes in occupational positions.^17^ However, the present analysis population may have been biased toward workers for large companies, living in urban areas.

Comparison of the prevalence of multimorbidity with that reported in a previous study may also highlight the characteristics of the present study. In a recent study by Saito et al.,^18^ analyzing the claims data of 20-74-year-old individuals insured by the Health Insurance Association for Architecture and Civil Engineering companies, the age-sex standardized prevalence of multimorbidity across 15 chronic diseases was 26.1%. In the present analysis population, 20 412 (65.9%) had 2 or more target diseases. Compared with this previous study, the present analysis focused on current workers with a narrower age range (19-64 years). The most notable contrast may lie in the inclusion of a larger number of target diseases, covering broader categories such as gastrointestinal disorders, skin diseases, and psychiatric disorders. This broader scope may provide a more comprehensive picture of workers’ health problems in Japan. Nevertheless, given the differences in aims and methods, caution should be exercised when interpreting the results between the studies. Furthermore, the results demonstrated that the mean number of diseases was slightly higher among females (3.4) than males (2.8). This difference may be partly explained by the inclusion of diseases often observed in women, such as menstrual and menopausal disorders. In addition, the prevalence of anemia^19^ and constipation,^20^ which was previously reported to be more common in women, was consistently higher in females than males in this study.

Importantly, the analysis of association between disease number and WPAI showed greater ORs for WPAI-GH scores >0% across domains and in both males and females. Based on these results, each incremental disease was associated with increased odds of experiencing work and daily life impairments to some degree. Although the ORs were modest, the cumulative burden of multiple diseases may be substantial, depending on the combination of comorbidities. Therefore, these results underscore the importance of comorbidity prevention and appropriate care for preexisting diseases. A previous study on migraines found that coexistence with certain diseases further reduced work productivity,^21^ supporting the present findings.

Logistic regression analysis demonstrated that WPAI-associated diseases varied by domain and differed according to sex, underscoring the complexity of multimorbidity and its association with WPAI. In males, migraine, depression, dysthymia, insomnia, and diarrheal disorders were consistently associated with impairment across all domains, whereas in females, associations were found for migraine, insomnia, and atopic dermatitis. Painful conditions (eg, back pain and neck stiffness), allergic diseases (eg, atopic dermatitis and bronchial asthma), anxiety disorder, and sensory organ diseases in males, and menstrual disorders, depression, allergic rhinitis, and tension headaches in females may be predominantly associated with reduced work performance, but without linking to absence. In addition, certain diseases, such as schizophrenia in males and diabetes mellitus in females, were associated with impairments in daily life but not work performance. Interestingly, atopic dermatitis was associated with the absence from work in females but not in males. These patterns suggest that a complex interplay between disease and sex underlies these associations and indicate that interventions should be tailored by disease and sex, with targeted support for workers with the high-risk diseases identified in this study.

Consistent with the crude percentages reported in the original study, the predicted probabilities after adjusting for coexisting diseases and conditions remained high for psychiatric disorders, such as depression and dysthymia, and migraine. These results suggest that these diseases may be associated with reduced work productivity, independent of comorbidities, and thus may require particular attention. Moreover, compared with the reported crude percentages, the predicted probability of having a WPAI-GH score >0% was particularly lower for psychiatric disorders. This finding could indicate both independent and cumulative associations when they coexist with other diseases. Therefore, comprehensive health management that addresses both psychiatric disorders and comorbidities may serve as a cornerstone for improving overall health.

Subgroup analysis by age resulted in a very small number of participants with certain diseases such as dysthymia, which may make it difficult to delineate age-related trends in the predicted probability. However, some diseases suggest age-related variations in the predicted probability; for example, a temporary peak in the 40s for tension headaches in females. Associations between a disease and WPAI may be influenced by unmeasured factors, including hormonal shifts, changes in occupational and social positions, and role transitions in the life course, including early retirement. The predicted probability of some diseases such as depression, diabetes, and migraine in women, peaked among those aged 29 years and younger and declined with age. These findings should not be interpreted as a reduction in disease burden with age; rather, as previously reported, it is necessary to consider their association with retirement. It is important to consider the prevalence of each disease when interpreting these results. From a broader public health perspective, rare conditions with a high predicted probability of having WPAI such as femoral neck fractures, could be associated with a modest societal burden. In contrast, common diseases with only a modest probability of WPAI, such as allergic rhinitis, which was found to be the most common comorbidity combination pattern, including a single disease could be related to cumulative productivity loss at the organizational or societal level owing to its high prevalence.

This study provided a broad overview of the associations between worker health and productivity in a real world setting and might help highlight groups with a higher likelihood of productivity loss. The results revealed various patterns including common diseases with a lower likelihood of productivity loss and rarer conditions with a higher likelihood of productivity loss, with these patterns varying according to sex and age. These findings may provide preliminary information for priority setting, highlighting the importance of targeting sex- and age-specific groups when designing workplace health promotion programs (such as disease awareness, monitoring, encouraging medical consultation, and follow-ups) by occupational health staff and policymakers.

Nevertheless, derived from the same dataset, the present analysis shares common limitations with the original analysis.^11^ First, because this study was cross-sectional, causal relationships could not be established, and reverse causality could not be ruled out. For example, a loss of work productivity may have prompted health care–seeking behaviors, increasing the likelihood of receiving a diagnosis. Second, as the present study included survey respondents who were currently working, interpretation of the results might need to consider the “healthy worker effect.” Requirements for registration with the online health application may have introduced a bias toward workers with higher health consciousness and digital literacy. Additionally, the study population consisted mainly of employees of large enterprises. Therefore, the study population reflected a specific subgroup of Japanese workers. Compared with Japanese national labor force survey demographics,^17^ this study population had a greater proportion of males (present study: 72.0% vs labor force survey: 55.8%) and workers aged 50-64 years, particularly among males (54.1% vs 32.7%) (Table S3). These differences suggest that the present findings may not be generalizable to the broader working population in Japan. Third, claims data generated for reimbursement purposes may not accurately reflect clinical diagnoses, whereas survey responses are inherently subject to self-reporting bias.

From an analytical perspective, WPAI score dichotomization can result in a loss of information on the magnitude of work productivity impairment. The selection biases mentioned above may have partially contributed to the observed concentration of WPAI scores at 0, resulting in a non-normal, right-skewed distribution of positive values with limited variability in impairment severity, thereby limiting the stability of severity analyses. Future studies that include workers with greater variability across impairment levels should consider modeling impairment severity to better elucidate associations between health and productivity of workers. This analysis may not fully account for potential confounders including disease severity and control status, because such information was unavailable in the claims or survey data. As more severe or less-controlled diseases are likely to be associated with greater productivity losses, the observed associations with specific diseases or disease counts may partly reflect these unmeasured factors. For example, for diseases where comorbidity rates are associated with disease severity such as migraines with depression or neck stiffness, the observed results might indicate the underlying severity rather than the number of comorbidities. Moreover, our simultaneous inclusion of 44 target diseases without categorizing those by disease type or comorbidity patterns might introduce multicollinearity risk in the correlated diseases. Caution is thus warranted when interpreting the results. Finally, detailed work-related information including workload and workplace environment was not available in the database, and its association with work productivity impairment could not be assessed. Integrating clinical and occupational information would be helpful in clarifying the combined impact of multimorbidity and working conditions on productivity.

## 5. Conclusions

An increase in the number of diseases was significantly associated with impaired work productivity. Independent of comorbidities, psychiatric disorders and migraine were associated with higher odds of work productivity impairment, with sex- and age-related variations in the pattern of associated diseases. These findings highlight the importance of optimal and tailored management of preexisting conditions and the prevention of comorbidities. While psychiatric disorders and chronic painful conditions appear to be central to worker health management, attention should also be paid to common diseases with a modest probability of work productivity impairment to support the overall sustainability and improvement of work productivity.

## Supplementary Material

20260601_WPAI2_fnl_Supplemetary_rev02_fnl_clean_uiag033

## Data Availability

The data underlying this article will be shared on reasonable request to the corresponding author.
