# Supplementary material for: Associations of diverse diseases with work productivity among Japanese workers in the context of multimorbidity: additional analysis of a database study
Source: J Occup Health. 2026 Jun 27;68(1):uiag033. doi: 10.1093/joccuh/uiag033 (PMC13464672; doi:10.1093/joccuh/uiag033)
Supplement: Supplementary file 1 [file 20260601_wpai2_fnl_supplemetary_rev02_fnl_clean_uiag033.docx]

# Supplementary Figure 1. Frequencies of comorbidity combination patterns in males

The matrix shows the top 40 combination pattern of the coexisting 41 diseases, with black dots (present) and gray dots (absent). When multiple diseases coexisted, the corresponding black dots are connected by black lines. The bar chart at the top shows the size of the intersections (i.e., the pattern of comorbidity combinations), and the bar chart on the left shows the size of each disease. To create this plot, oral-related diseases, sensory organ diseases, and disorders of refraction and accommodation were excluded from the 44 target diseases.

A: Back pain, B: Migraine, C: Hearing loss, D: Iron-deficiency anemia, E: Depression, F: Diabetes mellitus, G: Anxiety disorders, H: Femoral neck fracture, I: COPD, J: Osteoarthritis, K: Acne vulgaris, M: Schizophrenia, N: Bronchial asthma, O: Ischemic stroke, P: Atopic dermatitis, Q: Dysthymia, R: Alcohol use disorder, S: Bipolar disorder, U: Epilepsy, V: Diarrheal disorders, W: Tension headache, X: Ischemic heart disease, Z: Neck stiffness, AA: Diseases of esophagus, stomach and duodenum, AB: Constipation, AC: Obesity, AD: Hypertension, AE: Hyperlipidemia, AF: Infectious diseases, AG: Pollinosis, AH: Menstrual disorders, AI: Menopausal disorders, AJ: Uterine cancer, AK: Breast cancer, AL: Prostate cancer, AM: Colon cancer, AN: Gastric cancer, AO: Liver cancer, AP: Lung cancer, AQ: Insomnia, AR: Allergic rhinitis

# Supplementary Figure 2. Frequencies of comorbidity combination patterns in females

The matrix shows the top 40 combination pattern of the coexisting 41 diseases, with black dots (present) and gray dots (absent). When multiple diseases coexisted, the corresponding black dots are connected by black lines. The vertical bar chart at the top shows the size of the intersections (i.e., the pattern of comorbidity combinations), and the horizontal bar chart on the left shows the size of each disease. To create this plot, oral-related diseases, sensory organ diseases, and disorders of refraction and accommodation were excluded from the 44 target diseases.

A: Back pain, B: Migraine, C: Hearing loss, D: Iron-deficiency anemia, E: Depression, F: Diabetes mellitus, G: Anxiety disorders, H: Femoral neck fracture, I: COPD, J: Osteoarthritis, K: Acne vulgaris, M: Schizophrenia, N: Bronchial asthma, O: Ischemic stroke, P: Atopic dermatitis, Q: Dysthymia, R: Alcohol use disorder, S: Bipolar disorder, U: Epilepsy, V: Diarrheal disorders, W: Tension headache, X: Ischemic heart disease, Z: Neck stiffness, AA: Diseases of esophagus, stomach and duodenum, AB: Constipation, AC: Obesity, AD: Hypertension, AE: Hyperlipidemia, AF: Infectious diseases, AG: Pollinosis, AH: Menstrual disorders, AI: Menopausal disorders, AJ: Uterine cancer, AK: Breast cancer, AL: Prostate cancer, AM: Colon cancer, AN: Gastric cancer, AO: Liver cancer, AP: Lung cancer, AQ: Insomnia, AR: Allergic rhinitis

# Supplementary Figure 3. Predicted probability of WPAI-GH score of >0% by age groups, for diseases significantly associated with WPAI score of >0% in males

The figure presents the predicted probabilities of WPAI scores >0% for diseases that were significantly associated with increased odds in the model for each sex.

WPAI-GH, WPAI questionnaire-General Health

# Supplementary Figure 4. Predicted probability of WPAI-GH score of >0% by age groups, for diseases significantly associated with WPAI score of >0% in females

The figure presents the predicted probabilities of WPAI scores >0% for diseases that were significantly associated with increased odds in the model for each sex.

WPAI-GH, WPAI questionnaire-General Health

# Supplementary Table 1. Sensitivity analyses using ordinal logistic regression models (0%, >0%–10%, >10%–30%, >30%) for association between diseases and WPAI score in males

(A) Absenteeism

| **Diseases** | **OR** | **95%CI** | **p value** |
| --- | --- | --- | --- |
| Back pain | 1.169 | 0.981–1.393 | 0.0811 |
| Migraine | 1.801 | 1.265–2.564 | 0.0011 |
| Hearing loss | 1.205 | 0.878–1.654 | 0.2490 |
| Iron-deficiency anemia | 1.111 | 0.738–1.673 | 0.6131 |
| Depression | 1.831 | 1.451–2.312 | <.0001 |
| Diabetes mellitus | 1.074 | 0.892–1.294 | 0.4504 |
| Anxiety disorders | 1.166 | 0.880–1.545 | 0.2839 |
| Femoral neck fracture | 3.631 | 0.429–30.712 | 0.2365 |
| COPD | 1.202 | 0.594–2.435 | 0.6090 |
| Osteoarthritis | 1.117 | 0.829–1.506 | 0.4665 |
| Acne vulgaris | 0.889 | 0.622–1.270 | 0.5174 |
| Disorders of refraction and accommodation | 0.833 | 0.686–1.011 | 0.0641 |
| Schizophrenia | 1.084 | 0.659–1.786 | 0.7499 |
| Bronchial asthma | 1.013 | 0.810–1.266 | 0.9121 |
| Ischemic stroke | 1.500 | 0.952–2.363 | 0.0805 |
| Atopic dermatitis | 0.911 | 0.682–1.217 | 0.5276 |
| Dysthymia | 2.057 | 1.087–3.893 | 0.0268 |
| Alcohol use disorder | 3.066 | 1.163–8.080 | 0.0235 |
| Bipolar disorder | 1.305 | 0.818–2.084 | 0.2641 |
| Oral-related diseases | 1.010 | 0.902–1.131 | 0.8639 |
| Epilepsy | 1.557 | 0.891–2.721 | 0.1197 |
| Diarrheal disorders | 1.631 | 1.357–1.959 | <.0001 |
| Tension headache | 1.151 | 0.624–2.122 | 0.6522 |
| Ischemic heart disease | 0.836 | 0.335–2.089 | 0.7019 |
| Sensory organ diseases | 1.173 | 0.979–1.406 | 0.0833 |
| Neck stiffness | 1.101 | 0.623–1.946 | 0.7408 |
| Diseases of esophagus, stomach and duodenum | 1.443 | 1.265–1.646 | <.0001 |
| Constipation | 1.435 | 1.159–1.776 | 0.0009 |
| Obesity | 0.536 | 0.194–1.477 | 0.2277 |
| Hypertension | 1.104 | 0.951–1.280 | 0.1933 |
| Hyperlipidemia | 1.009 | 0.871–1.170 | 0.9048 |
| Infectious diseases | 1.114 | 0.943–1.316 | 0.2055 |
| Pollinosis | 0.681 | 0.423–1.096 | 0.1132 |
| Prostate cancer | 1.566 | 0.753–3.254 | 0.2299 |
| Colon cancer | 0.904 | 0.416–1.962 | 0.7980 |
| Gastric cancer | 1.835 | 0.852–3.952 | 0.1211 |
| Liver cancer | 1.154 | 0.258–5.149 | 0.8515 |
| Lung cancer | 0.198 | 0.025–1.557 | 0.1238 |
| Insomnia | 1.421 | 1.151–1.753 | 0.0011 |
| Allergic rhinitis | 0.999 | 0.865–1.154 | 0.9902 |

(B) Presenteeism

| **Diseases** | **OR** | **95%CI** | **p value** |
| --- | --- | --- | --- |
| Back pain | 1.187 | 1.080–1.305 | 0.0004 |
| Migraine | 1.701 | 1.360–2.128 | <.0001 |
| Hearing loss | 1.044 | 0.876–1.246 | 0.6287 |
| Iron-deficiency anemia | 1.244 | 0.981–1.578 | 0.0720 |
| Depression | 1.970 | 1.709–2.271 | <.0001 |
| Diabetes mellitus | 0.996 | 0.902–1.100 | 0.9393 |
| Anxiety disorders | 1.437 | 1.215–1.699 | <.0001 |
| Femoral neck fracture | 3.138 | 0.643–15.312 | 0.1574 |
| COPD | 0.903 | 0.595–1.373 | 0.6339 |
| Osteoarthritis | 0.986 | 0.839–1.158 | 0.8594 |
| Acne vulgaris | 1.160 | 0.982–1.369 | 0.0805 |
| Disorders of refraction and accommodation | 0.945 | 0.857–1.043 | 0.2605 |
| Schizophrenia | 1.371 | 0.985–1.908 | 0.0611 |
| Bronchial asthma | 1.250 | 1.116–1.399 | 0.0001 |
| Ischemic stroke | 1.117 | 0.841–1.483 | 0.4465 |
| Atopic dermatitis | 1.211 | 1.059–1.384 | 0.0050 |
| Dysthymia | 2.172 | 1.372–3.440 | 0.0009 |
| Alcohol use disorder | 1.016 | 0.459–2.246 | 0.9692 |
| Bipolar disorder | 1.311 | 0.951–1.808 | 0.0982 |
| Oral-related diseases | 0.960 | 0.910–1.012 | 0.1316 |
| Epilepsy | 1.301 | 0.910–1.859 | 0.1491 |
| Diarrheal disorders | 1.222 | 1.096–1.362 | 0.0003 |
| Tension headache | 1.550 | 1.090–2.205 | 0.0148 |
| Ischemic heart disease | 0.889 | 0.524–1.508 | 0.6620 |
| Sensory organ diseases | 1.120 | 1.022–1.228 | 0.0155 |
| Neck stiffness | 1.419 | 1.018–1.980 | 0.0392 |
| Diseases of esophagus, stomach and duodenum | 1.058 | 0.988–1.132 | 0.1054 |
| Constipation | 1.146 | 1.007–1.303 | 0.0383 |
| Obesity | 0.710 | 0.458–1.102 | 0.1272 |
| Hypertension | 0.901 | 0.834–0.973 | 0.0077 |
| Hyperlipidemia | 0.929 | 0.862–1.001 | 0.0531 |
| Infectious diseases | 0.951 | 0.871–1.037 | 0.2526 |
| Pollinosis | 0.939 | 0.760–1.162 | 0.5637 |
| Menopausal disorders | 1.736 | 0.606–4.970 | 0.3040 |
| Prostate cancer | 0.878 | 0.553–1.392 | 0.5796 |
| Colon cancer | 0.952 | 0.638–1.422 | 0.8108 |
| Gastric cancer | 0.908 | 0.534–1.546 | 0.7230 |
| Liver cancer | 1.155 | 0.473–2.823 | 0.7518 |
| Lung cancer | 1.225 | 0.673–2.231 | 0.5064 |
| Insomnia | 1.568 | 1.387–1.772 | <.0001 |
| Allergic rhinitis | 1.004 | 0.935–1.079 | 0.9055 |

(C) Total work impairment

| **Diseases** | **OR** | **95%CI** | **p value** |
| --- | --- | --- | --- |
| Back pain | 1.186 | 1.080–1.303 | 0.0004 |
| Migraine | 1.630 | 1.302–2.039 | <.0001 |
| Hearing loss | 1.051 | 0.883–1.252 | 0.5746 |
| Iron-deficiency anemia | 1.235 | 0.975–1.564 | 0.0795 |
| Depression | 1.971 | 1.710–2.273 | <.0001 |
| Diabetes mellitus | 1.031 | 0.934–1.137 | 0.5434 |
| Anxiety disorders | 1.448 | 1.225–1.712 | <.0001 |
| Femoral neck fracture | 2.717 | 0.555–13.304 | 0.2174 |
| COPD | 0.946 | 0.627–1.425 | 0.7890 |
| Osteoarthritis | 0.989 | 0.843–1.160 | 0.8893 |
| Acne vulgaris | 1.128 | 0.956–1.331 | 0.1546 |
| Disorders of refraction and accommodation | 0.919 | 0.833–1.012 | 0.0872 |
| Schizophrenia | 1.258 | 0.901–1.757 | 0.1773 |
| Bronchial asthma | 1.215 | 1.085–1.360 | 0.0007 |
| Ischemic stroke | 1.224 | 0.926–1.617 | 0.1552 |
| Atopic dermatitis | 1.174 | 1.028–1.341 | 0.0183 |
| Dysthymia | 2.193 | 1.380–3.484 | 0.0009 |
| Alcohol use disorder | 1.149 | 0.523–2.522 | 0.7293 |
| Bipolar disorder | 1.372 | 0.991–1.899 | 0.0567 |
| Oral-related diseases | 0.976 | 0.925–1.029 | 0.3668 |
| Epilepsy | 1.377 | 0.966–1.962 | 0.0772 |
| Diarrheal disorders | 1.295 | 1.163–1.442 | <.0001 |
| Tension headache | 1.509 | 1.061–2.148 | 0.0222 |
| Ischemic heart disease | 0.912 | 0.536–1.551 | 0.7326 |
| Sensory organ diseases | 1.142 | 1.042–1.251 | 0.0043 |
| Neck stiffness | 1.342 | 0.962–1.870 | 0.0829 |
| Diseases of esophagus, stomach and duodenum | 1.085 | 1.014–1.161 | 0.0175 |
| Constipation | 1.182 | 1.041–1.343 | 0.0101 |
| Obesity | 0.611 | 0.391–0.953 | 0.0300 |
| Hypertension | 0.915 | 0.848–0.987 | 0.0216 |
| Hyperlipidemia | 0.923 | 0.857–0.994 | 0.0341 |
| Infectious diseases | 0.961 | 0.882–1.047 | 0.3670 |
| Pollinosis | 0.882 | 0.714–1.090 | 0.2452 |
| Menopausal disorders | 1.536 | 0.535–4.408 | 0.4253 |
| Prostate cancer | 0.941 | 0.599–1.478 | 0.7917 |
| Colon cancer | 0.893 | 0.598–1.332 | 0.5789 |
| Gastric cancer | 1.105 | 0.660–1.849 | 0.7048 |
| Liver cancer | 1.147 | 0.475–2.770 | 0.7607 |
| Lung cancer | 1.115 | 0.613–2.029 | 0.7209 |
| Insomnia | 1.578 | 1.397–1.783 | <.0001 |
| Allergic rhinitis | 1.012 | 0.942–1.086 | 0.7462 |

(D) Total activity impairment

| **Diseases** | **OR** | **95%CI** | **p value** |
| --- | --- | --- | --- |
| Back pain | 1.218 | 1.109–1.338 | <.0001 |
| Migraine | 1.775 | 1.421–2.217 | <.0001 |
| Hearing loss | 1.090 | 0.915–1.297 | 0.3349 |
| Iron-deficiency anemia | 1.195 | 0.943–1.515 | 0.1405 |
| Depression | 1.873 | 1.625–2.159 | <.0001 |
| Diabetes mellitus | 0.997 | 0.903–1.100 | 0.9473 |
| Anxiety disorders | 1.425 | 1.206–1.684 | <.0001 |
| Femoral neck fracture | 2.851 | 0.585–13.897 | 0.1949 |
| COPD | 0.953 | 0.631–1.440 | 0.8192 |
| Osteoarthritis | 1.120 | 0.957–1.310 | 0.1594 |
| Acne vulgaris | 1.089 | 0.922–1.286 | 0.3165 |
| Disorders of refraction and accommodation | 0.940 | 0.853–1.036 | 0.2144 |
| Schizophrenia | 1.583 | 1.139–2.200 | 0.0063 |
| Bronchial asthma | 1.237 | 1.106–1.385 | 0.0002 |
| Ischemic stroke | 1.190 | 0.900–1.574 | 0.2214 |
| Atopic dermatitis | 1.168 | 1.022–1.335 | 0.0228 |
| Dysthymia | 1.974 | 1.249–3.121 | 0.0036 |
| Alcohol use disorder | 1.023 | 0.463–2.260 | 0.9554 |
| Bipolar disorder | 1.133 | 0.822–1.563 | 0.4459 |
| Oral-related diseases | 0.952 | 0.902–1.004 | 0.0678 |
| Epilepsy | 1.319 | 0.925–1.882 | 0.1263 |
| Diarrheal disorders | 1.171 | 1.051–1.306 | 0.0043 |
| Tension headache | 1.349 | 0.947–1.922 | 0.0973 |
| Ischemic heart disease | 0.827 | 0.487–1.405 | 0.4821 |
| Sensory organ diseases | 1.128 | 1.030–1.236 | 0.0097 |
| Neck stiffness | 1.503 | 1.081–2.091 | 0.0154 |
| Diseases of esophagus, stomach and duodenum | 1.057 | 0.988–1.131 | 0.1094 |
| Constipation | 1.119 | 0.984–1.272 | 0.0865 |
| Obesity | 0.763 | 0.497–1.172 | 0.2169 |
| Hypertension | 0.914 | 0.847–0.986 | 0.0200 |
| Hyperlipidemia | 0.937 | 0.870–1.009 | 0.0858 |
| Infectious diseases | 0.994 | 0.913–1.083 | 0.8976 |
| Pollinosis | 0.983 | 0.798–1.212 | 0.8754 |
| Menopausal disorders | 2.271 | 0.810–6.367 | 0.1189 |
| Prostate cancer | 1.035 | 0.663–1.617 | 0.8790 |
| Colon cancer | 1.030 | 0.694–1.527 | 0.8847 |
| Gastric cancer | 0.982 | 0.582–1.657 | 0.9466 |
| Liver cancer | 0.898 | 0.358–2.250 | 0.8179 |
| Lung cancer | 0.963 | 0.522–1.775 | 0.9030 |
| Insomnia | 1.494 | 1.322–1.689 | <.0001 |
| Allergic rhinitis | 1.020 | 0.950–1.096 | 0.5759 |

Sensitivity analyses were performed using ordinal logistic regression models with alternative categorization of WPAI scores*.*

CI, confidence interval; COPD, chronic obstructive pulmonary disease; OR, odds ratio; WPAI-GH, WPAI questionnaire-General Health

# Supplementary Table 2. Sensitivity analyses using ordinal logistic regression models (0%, >0%–10%, >10%–30%, >30%) for association between diseases and WPAI score in females

(A) Absenteeism

| **Diseases** | **OR** | **95%CI** | **p value** |
| --- | --- | --- | --- |
| Back pain | 0.918 | 0.706–1.195 | 0.5268 |
| Migraine | 1.829 | 1.274–2.625 | 0.0011 |
| Hearing loss | 1.229 | 0.795–1.899 | 0.3530 |
| Iron-deficiency anemia | 1.087 | 0.822–1.436 | 0.5589 |
| Depression | 1.391 | 0.934–2.072 | 0.1049 |
| Diabetes mellitus | 1.088 | 0.750–1.577 | 0.6565 |
| Anxiety disorders | 0.966 | 0.651–1.433 | 0.8632 |
| COPD | 1.555 | 0.409–5.913 | 0.5168 |
| Osteoarthritis | 1.302 | 0.905–1.875 | 0.1551 |
| Acne vulgaris | 1.332 | 0.993–1.787 | 0.0559 |
| Disorders of refraction and accommodation | 0.879 | 0.677–1.142 | 0.3341 |
| Schizophrenia | 1.036 | 0.473–2.269 | 0.9300 |
| Bronchial asthma | 1.156 | 0.863–1.546 | 0.3309 |
| Ischemic stroke | 0.208 | 0.028–1.564 | 0.1272 |
| Atopic dermatitis | 1.322 | 0.980–1.783 | 0.0680 |
| Dysthymia | 0.804 | 0.192–3.371 | 0.7654 |
| Bipolar disorder | 0.938 | 0.384–2.289 | 0.8879 |
| Oral-related diseases | 1.023 | 0.861–1.216 | 0.7936 |
| Epilepsy | 0.635 | 0.242–1.666 | 0.3563 |
| Diarrheal disorders | 1.710 | 1.317–2.221 | <.0001 |
| Tension headache | 1.670 | 0.894–3.118 | 0.1075 |
| Ischemic heart disease | 2.212 | 0.214–22.845 | 0.5050 |
| Sensory organ diseases | 0.963 | 0.741–1.250 | 0.7760 |
| Neck stiffness | 1.146 | 0.666–1.974 | 0.6224 |
| Diseases of esophagus, stomach and duodenum | 1.305 | 1.071–1.588 | 0.0082 |
| Constipation | 1.180 | 0.913–1.526 | 0.2059 |
| Obesity | 1.644 | 0.700–3.864 | 0.2541 |
| Hypertension | 1.143 | 0.851–1.537 | 0.3741 |
| Hyperlipidemia | 0.775 | 0.594–1.010 | 0.0597 |
| Infectious diseases | 0.972 | 0.774–1.220 | 0.8042 |
| Pollinosis | 0.993 | 0.542–1.821 | 0.9831 |
| Menstrual disorders | 1.108 | 0.828–1.483 | 0.4903 |
| Menopausal disorders | 1.128 | 0.815–1.561 | 0.4691 |
| Uterine cancer | 0.574 | 0.179–1.845 | 0.3517 |
| Breast cancer | 1.604 | 1.038–2.480 | 0.0335 |
| Colon cancer | 1.659 | 0.576–4.777 | 0.3483 |
| Gastric cancer | 3.764 | 0.944–15.007 | 0.0603 |
| Insomnia | 1.459 | 1.064–2.001 | 0.0191 |
| Allergic rhinitis | 1.003 | 0.816–1.233 | 0.9746 |

(B) Presenteeism

| **Diseases** | **OR** | **95%CI** | **p value** |
| --- | --- | --- | --- |
| Back pain | 1.025 | 0.892–1.178 | 0.7277 |
| Migraine | 1.721 | 1.368–2.166 | <.0001 |
| Hearing loss | 1.245 | 0.985–1.574 | 0.0670 |
| Iron-deficiency anemia | 0.918 | 0.790–1.066 | 0.2635 |
| Depression | 1.867 | 1.474–2.365 | <.0001 |
| Diabetes mellitus | 1.232 | 1.011–1.501 | 0.0386 |
| Anxiety disorders | 1.122 | 0.899–1.402 | 0.3093 |
| COPD | 0.697 | 0.285–1.704 | 0.4287 |
| Osteoarthritis | 1.027 | 0.837–1.260 | 0.7981 |
| Acne vulgaris | 1.178 | 1.001–1.387 | 0.0488 |
| Disorders of refraction and accommodation | 1.121 | 0.981–1.282 | 0.0938 |
| Schizophrenia | 0.881 | 0.535–1.452 | 0.6189 |
| Bronchial asthma | 1.000 | 0.853–1.173 | 0.9979 |
| Ischemic stroke | 0.572 | 0.313–1.045 | 0.0694 |
| Atopic dermatitis | 1.222 | 1.037–1.439 | 0.0168 |
| Dysthymia | 0.521 | 0.238–1.138 | 0.1017 |
| Bipolar disorder | 1.152 | 0.675–1.966 | 0.6029 |
| Oral-related diseases | 0.915 | 0.841–0.995 | 0.0385 |
| Epilepsy | 1.224 | 0.737–2.033 | 0.4339 |
| Diarrheal disorders | 1.201 | 1.026–1.407 | 0.0230 |
| Tension headache | 1.669 | 1.127–2.471 | 0.0105 |
| Ischemic heart disease | 2.070 | 0.423–10.141 | 0.3695 |
| Sensory organ diseases | 0.971 | 0.849–1.111 | 0.6709 |
| Neck stiffness | 0.954 | 0.685–1.330 | 0.7822 |
| Diseases of esophagus, stomach and duodenum | 1.152 | 1.040–1.277 | 0.0069 |
| Constipation | 1.051 | 0.911–1.213 | 0.4938 |
| Obesity | 0.826 | 0.468–1.456 | 0.5083 |
| Hypertension | 0.877 | 0.747–1.028 | 0.1061 |
| Hyperlipidemia | 0.785 | 0.687–0.896 | 0.0003 |
| Infectious diseases | 1.022 | 0.910–1.149 | 0.7112 |
| Pollinosis | 1.196 | 0.869–1.644 | 0.2717 |
| Menstrual disorders | 1.436 | 1.232–1.674 | <.0001 |
| Menopausal disorders | 0.848 | 0.706–1.018 | 0.0770 |
| Uterine cancer | 1.229 | 0.734–2.059 | 0.4326 |
| Breast cancer | 0.663 | 0.501–0.878 | 0.0041 |
| Colon cancer | 1.066 | 0.548–2.073 | 0.8515 |
| Gastric cancer | 1.241 | 0.396–3.887 | 0.7105 |
| Lung cancer | 0.290 | 0.034–2.487 | 0.2590 |
| Insomnia | 1.450 | 1.207–1.743 | <.0001 |
| Allergic rhinitis | 1.148 | 1.034–1.274 | 0.0095 |

(C) Total work impairment

| **Diseases** | **OR** | **95%CI** | **p value** |
| --- | --- | --- | --- |
| Back pain | 1.034 | 0.900–1.188 | 0.6341 |
| Migraine | 1.743 | 1.383–2.196 | <.0001 |
| Hearing loss | 1.279 | 1.009–1.620 | 0.0417 |
| Iron-deficiency anemia | 0.943 | 0.813–1.094 | 0.4386 |
| Depression | 1.833 | 1.442–2.330 | <.0001 |
| Diabetes mellitus | 1.200 | 0.985–1.461 | 0.0702 |
| Anxiety disorders | 1.136 | 0.909–1.420 | 0.2619 |
| COPD | 0.629 | 0.250–1.581 | 0.3241 |
| Osteoarthritis | 1.109 | 0.905–1.360 | 0.3190 |
| Acne vulgaris | 1.180 | 1.003–1.389 | 0.0462 |
| Disorders of refraction and accommodation | 1.132 | 0.991–1.294 | 0.0676 |
| Schizophrenia | 1.038 | 0.625–1.722 | 0.8856 |
| Bronchial asthma | 1.024 | 0.874–1.200 | 0.7700 |
| Ischemic stroke | 0.519 | 0.282–0.952 | 0.0341 |
| Atopic dermatitis | 1.229 | 1.043–1.448 | 0.0138 |
| Dysthymia | 0.503 | 0.228–1.110 | 0.0887 |
| Bipolar disorder | 0.951 | 0.552–1.639 | 0.8566 |
| Oral-related diseases | 0.930 | 0.855–1.011 | 0.0893 |
| Epilepsy | 1.190 | 0.716–1.979 | 0.5026 |
| Diarrheal disorders | 1.292 | 1.104–1.513 | 0.0014 |
| Tension headache | 1.830 | 1.231–2.721 | 0.0028 |
| Ischemic heart disease | 1.852 | 0.378–9.070 | 0.4469 |
| Sensory organ diseases | 0.928 | 0.811–1.061 | 0.2723 |
| Neck stiffness | 0.905 | 0.649–1.262 | 0.5562 |
| Diseases of esophagus, stomach and duodenum | 1.133 | 1.023–1.256 | 0.0167 |
| Constipation | 1.055 | 0.915–1.217 | 0.4634 |
| Obesity | 1.040 | 0.598–1.810 | 0.8899 |
| Hypertension | 0.907 | 0.774–1.063 | 0.2270 |
| Hyperlipidemia | 0.796 | 0.698–0.908 | 0.0007 |
| Infectious diseases | 1.019 | 0.908–1.145 | 0.7451 |
| Pollinosis | 1.166 | 0.845–1.609 | 0.3492 |
| Menstrual disorders | 1.422 | 1.219–1.658 | <.0001 |
| Menopausal disorders | 0.864 | 0.721–1.037 | 0.1163 |
| Uterine cancer | 1.159 | 0.694–1.936 | 0.5738 |
| Breast cancer | 0.737 | 0.560–0.970 | 0.0297 |
| Colon cancer | 1.200 | 0.624–2.307 | 0.5842 |
| Gastric cancer | 1.624 | 0.526–5.014 | 0.3995 |
| Lung cancer | 0.265 | 0.031–2.279 | 0.2263 |
| Insomnia | 1.417 | 1.178–1.704 | 0.0002 |
| Allergic rhinitis | 1.176 | 1.060–1.305 | 0.0022 |

(D) Total activity impairment

| **Diseases** | **OR** | **95%CI** | **p value** |
| --- | --- | --- | --- |
| Back pain | 1.066 | 0.929–1.222 | 0.3642 |
| Migraine | 1.638 | 1.302–2.060 | <.0001 |
| Hearing loss | 1.162 | 0.920–1.467 | 0.2085 |
| Iron-deficiency anemia | 0.967 | 0.834–1.120 | 0.6536 |
| Depression | 1.759 | 1.389–2.227 | <.0001 |
| Diabetes mellitus | 1.308 | 1.077–1.590 | 0.0069 |
| Anxiety disorders | 1.247 | 1.000–1.554 | 0.0496 |
| COPD | 0.627 | 0.257–1.530 | 0.3049 |
| Osteoarthritis | 1.156 | 0.946–1.413 | 0.1576 |
| Acne vulgaris | 1.058 | 0.899–1.245 | 0.4977 |
| Disorders of refraction and accommodation | 1.057 | 0.926–1.206 | 0.4149 |
| Schizophrenia | 1.200 | 0.728–1.979 | 0.4743 |
| Bronchial asthma | 1.002 | 0.856–1.174 | 0.9767 |
| Ischemic stroke | 0.519 | 0.286–0.942 | 0.0310 |
| Atopic dermatitis | 1.268 | 1.078–1.492 | 0.0042 |
| Dysthymia | 0.764 | 0.364–1.607 | 0.4785 |
| Bipolar disorder | 1.478 | 0.863–2.530 | 0.1548 |
| Oral-related diseases | 0.924 | 0.850–1.004 | 0.0618 |
| Epilepsy | 1.131 | 0.680–1.883 | 0.6349 |
| Diarrheal disorders | 1.168 | 0.998–1.366 | 0.0528 |
| Tension headache | 1.785 | 1.207–2.641 | 0.0037 |
| Ischemic heart disease | 1.767 | 0.360–8.657 | 0.4828 |
| Sensory organ diseases | 1.004 | 0.879–1.147 | 0.9563 |
| Neck stiffness | 0.782 | 0.561–1.091 | 0.1475 |
| Diseases of esophagus, stomach and duodenum | 1.179 | 1.065–1.305 | 0.0015 |
| Constipation | 1.097 | 0.952–1.263 | 0.2005 |
| Obesity | 1.018 | 0.590–1.759 | 0.9480 |
| Hypertension | 0.898 | 0.767–1.050 | 0.1783 |
| Hyperlipidemia | 0.780 | 0.684–0.889 | 0.0002 |
| Infectious diseases | 1.061 | 0.946–1.191 | 0.3115 |
| Pollinosis | 1.058 | 0.770–1.456 | 0.7269 |
| Menstrual disorders | 1.363 | 1.170–1.588 | <.0001 |
| Menopausal disorders | 0.960 | 0.803–1.148 | 0.6530 |
| Uterine cancer | 0.866 | 0.513–1.461 | 0.5886 |
| Breast cancer | 0.755 | 0.576–0.988 | 0.0409 |
| Colon cancer | 0.874 | 0.448–1.704 | 0.6920 |
| Gastric cancer | 1.324 | 0.426–4.112 | 0.6278 |
| Lung cancer | 0.242 | 0.028–2.094 | 0.1975 |
| Insomnia | 1.389 | 1.157–1.668 | 0.0004 |
| Allergic rhinitis | 1.111 | 1.002–1.232 | 0.0455 |

Sensitivity analyses were performed using ordinal logistic regression models with alternative categorization of WPAI scores*.*

CI, confidence interval; COPD, chronic obstructive pulmonary disease; OR, odds ratio; WPAI-GH, WPAI questionnaire-General Health

# Supplementary Table 3. Comparison of demographic characteristics of the present study population with the population from the Japanese national labor force survey

| **Characteristics** | | **Present study** |  | **Labor force survey^a^ (10,000 persons)** |
| --- | --- | --- | --- | --- |
|  |  | **n (%)** |  | **n (%)** |
| **Total, N** | | 30,953 |  | 5,627 |
| **Sex** | |  |  |  |
|  | Male | 22,292 (72.0) |  | 3,139 (55.8) |
|  | Female | 8,661 (28.0) |  | 2,488 (44.2) |
| **Age distribution within sex^b^** | | | | |
|  | **Male** |  |  |  |
|  | ≤29^c^ | 1,083 (4.9) |  | 505 (16.1) |
|  | 30–39 | 3,083 (13.8) |  | 711 (22.7) |
|  | 40–49 | 6,055 (27.2) |  | 896 (28.5) |
|  | 50–64 | 12,071 (54.1) |  | 1,027 (32.7) |
|  | **Female** |  |  |  |
|  | ≤29^c^ | 768 (8.9) |  | 450 (18.1) |
|  | 30–39 | 1,501 (17.3) |  | 535 (21.5) |
|  | 40–49 | 3,042 (35.1) |  | 714 (28.7) |
|  | 50–64 | 3,350 (38.7) |  | 789 (31.7) |

^a^ Data were obtained from the Japanese national labor force survey (<https://www.e-stat.go.jp/en/stat-search/files?page=1&stat_infid=000031702902>). These figures are displayed in units of 10,000 persons.

^b^ Percentages for age groups are calculated within each sex.

^c^ Aged ≤29 years refers to the 19–29 year-old group in this study, and the sum of 20–24 and 25–29 year-old groups in the Japanese national labor force survey.
